# Case of Cholera-Infantum with Anuria for Five Days

**Published:** 1871-02

**Authors:** C. W. Stevens

**Affiliations:** Charlestown, Mass.


### CASE OF CHOLERA INFANTUM WITH ANURIA FOR FIVE DAYS. RECOVERY.

By C. W. Stevens, M. D., Charlestown, Mass.

A child, aged one year, was taken suddenly, on Oct. 1st, 1870, with vomiting and diarrhœa. In the vomita were pieces of corned beef, which the mother had given to "harden her child." Oct. 4th, I was sent for, after the usual household remedies had been exhausted. Found the primary symptoms of Cheyne's hydrencephaloid disease. Not even a teaspoonful of water could be retained in the stomach, and there was an incessant diarrhœa of greenish mucus. Continual worrying. Ordered

| | |
|---|---|
| Acetat. plumbi. . . . | gr. v. |
| Acid acetic. | |
| Tinct. opii, . . . | aa gutt. v. |
| Glycerine, . . . | ʒ iss. |
| Aqua destill., . . . | ℥ i. M. |

One teaspoonful after vomiting.

One teaspoonful of cold barley water was to be taken occasionally, and an enema of one table-spoonful of mutton-broth every two hours.

Oct. 5th.—Had rejected both the barley-water and the lead mixture, but retained the enemata. Ordered

| | |
|---|---|
| Hydrarg. chlorid. mitis, . | gr. i. |
| Pulv. opii. . . . | gr. ¼. |
| Pulv. sacchari, . . . | gr. vii. Chart vi. |

One powder to be taken after vomiting. To take the froth of the white of egg and brandy nog.

Oct. 6th.—Second stage of spurious hydrocephalus—depressed fontanellæ, eyes sunken and rolled up, cold extremities, continual worrying, inability to rise from the cradle. Ordered hot bottles to feet and hot fomentations to abdomen.

Oct. 7th.—Has vomited only once or twice. Has passed no urine for two days. Ordered two drops of spts. of nit. ether in a teaspoonful of barley water every half hour.

Oct. 8th.—Continued anuria. That it is suppression of urine as in cholera, and not simple retention, I assured myself by percussion. Ordered fomentations of hot cloths over the hypogastrium, and

| | |
|---|---|
| Fol. buchu, . . . . | ʒ ij. |
| Sem. anisi, . . . . | ℈ i. |
| Aqua, . . . . | ℥ viij. |

One tablespoonful of the infusion every four hours.

Oct. 9th.—Stomach retains milk and water. Continued anuria. I gave at once six drops of paregoric, and ordered two drops to be given every half hour.

Oct. 10th.—The baby passed water for the first time in about four hours from the first dose of the paregoric. From this moment the child began rapidly to mend. I should add that mutton broth, oysters, and brandy and egg, had been regularly continued every day. I am of the opinion that the paregoric was the most efficient aid in causing micturition. I was led to use it from the utility of camphor and opium in retention of urine.

